# A novel e-learning tool to improve knowledge and awareness of pelvic radiotherapy late effects: qualitative responses amongst therapeutic radiographers

**DOI:** 10.1259/bjro.20210036

**Published:** 2021-10-28

**Authors:** Lauren Ann Oliver, Bridget Porritt, Mike Kirby

**Affiliations:** ^1^ University of Liverpool, Liverpool, UK

## Abstract

**Objectives:**

This study aimed to investigate the effectiveness of a novel e-learning intervention to increase knowledge, awareness and confidence surrounding pelvic radiotherapy late effects amongst therapeutic radiographers (RTTs), and to change staff perceptions of responsibility in providing such information to patients.

**Methods:**

The e-learning intervention was developed using blended learning software (Articulate Global, New York). 23 therapeutic radiographers within a single UK radiotherapy institution received the e-learning. Semi-structured interviews and questionnaires were utilised pre- and post-intervention to obtain qualitative and quantitative results. Thematic analysis of coded interview responses identified recurring themes, whilst statistical analysis was conducted using a Wilcoxon signed-rank test. This first paper presents the qualitative results.

**Results:**

Thematic analysis revealed increased knowledge and awareness of pelvic radiotherapy late effects amongst participants. Five key themes were identified: Knowledge/Confidence; Consent; Professional Responsibility; Gaps within Practice and Time/Space. Whilst several staff reported increased confidence in discussing late effects with patients, further training utilising “blended” pedagogical approaches may be required to achieve longstanding improvements. Following e-learning, participants demonstrated increased professional responsibility to deliver late effects information to patients.

**Conclusion:**

The novel e-learning intervention increased staff knowledge, awareness and confidence surrounding pelvic radiotherapy late effects, whilst changing staff perceptions on professional responsibility in delivering such information.

**Advances in knowledge:**

The e-learning has been disseminated to all hospitals within the region including a new “Radiotherapy Late Effects Clinic”, educating various healthcare professionals. Study recommendations have led to introduction of dedicated radiotherapy late effects modules on a novel MSc programme at a UK University.

## Background

Pelvic malignancies are amongst the most common cancers, with over 50% of diagnoses in 2017 including prostate and bowel cancers.^1^ One of the most effective treatment modalities for pelvic malignancies is radiotherapy, with significant advancements in treatment techniques over the last two decades.^2^ However, increased overall survival rates associated with radiotherapy must be considered alongside the growing prevalence of chronic post-treatment toxicities, commonly termed “late effects” (LEs). Radiation-induced tissue damage to surrounding pelvic structures results in LEs including gastrointestinal and genitourinary toxicities, chronic fatigue and sexual dysfunction. The true prevalence of pelvic radiotherapy LEs is not well established due to the delayed onset of symptoms up to decades post-treatment, and frequent underreporting attributed to a lack of awareness amongst many healthcare professionals (HCPs).^3^ An estimated 50% of pelvic radiotherapy patients experience chronic gastrointestinal symptoms including diarrhoea, fecal incontinence and rectal bleeding, which significantly impact their quality of life (QoL).^3,4^ Such LEs not only present a physical burden, but also affect survivors’ psychosocial, sexual and emotional wellbeing.

A national drive aims to improve aftercare for pelvic radiotherapy patients; recommendations by the National Health Service (NHS), Department of Health and National Institute for Health and Care Excellence (NICE) have stated the necessity for improved information provision for patients regarding radiotherapy LEs, empowering patients with knowledge to self-manage symptoms.^5,6^ Furthermore, the National Cancer Research Institute highlight informing patients of LEs as one of the UK’s top ten research priorities.^7^

Inadequate information on the LEs of pelvic radiotherapy is widely reported as a critical unmet need of cancer survivors,^8^ significantly impacting patients’ psychosocial and physical wellbeing.^9^ However, several studies report many HCPs do not recognise LE information disclosure as their responsibility; qualitative data reported by Griffiths et al^10^ demonstrated many staff view this as “the doctor’s job”, with a self-reported “lack of knowledge/education of LEs”.^10^ Such findings highlight a requirement for ongoing education focussed on radiotherapy LEs for staff post-registration, with therapeutic radiographers possessing a unique position to disclose such information due to the rapport built with patients throughout treatment.^11,12^

Traditional training possesses barriers including time constraints, limited staff/trainer availability and cost.^13^ An alternative approach that overcomes such barriers is e-learning, which permits training using an electronic medium.^14^ The flexible, asynchronous nature of e-learning allows remote access at any time,^15^ overcoming scheduling issues within busy clinical departments. E-learning is currently employed within healthcare to deliver mandatory training and continued professional development, a requirement of professional practice specified by the Health Care and Professions Council (HCPC) and Society and College of Radiographers (SCoR).^16,17^ In addition, Health Education England supports “*e-Learning for Healthcare”* to develop e-learning for health services, however this does not currently feature training on pelvic radiotherapy LEs.^18^

Furthermore, e-learning provides a robust solution for training as content can be updated online with changes in healthcare practice, reducing the need to issue multiple, new hardcopy resources. Inclusion of multiple-choice questions (MCQs), case studies and video narratives provide a dynamic learning experience as opposed to traditional passive pedagogical elements, facilitating critical thinking and development of long-term knowledge.^19^

This study aimed to investigate the effectiveness of a novel e-learning intervention to increase therapeutic radiographers’ knowledge, awareness and confidence surrounding the LEs of pelvic radiotherapy, and to investigate whether participants’ perceptions of their professional responsibility to provide such information changed post-intervention. Additionally, our methods are easily applicable to radiology and other healthcare professionals, permitting development of similar training/CPD interventions across the wider workforce. Here, we present the qualitative results of a mixed-methods study.

## Methods and material

The study was conducted within a single radiotherapy institution over a 12-month period. In order to determine the effectiveness of the novel e-learning intervention, a mixed-methods approach was utilised to obtain qualitative and quantitative data. Questionnaires were developed to ascertain changes in staff knowledge, awareness, confidence and perceptions of responsibility surrounding pelvic radiotherapy LEs pre- and post-intervention. The addition of pre- and post-intervention staff focus groups facilitated collection of more detailed views and opinions, whilst such data triangulation increased study validity.^20^

This study was undertaken as an MSc project following service development guidelines. Research and innovation approval was granted within the clinical department, whilst ethical approval was granted by the University of Liverpool due to involvement of human participants.

### Recruitment procedure

A study description, participant information sheets and consent forms were emailed to all band 4–6 therapeutic radiographers working in treatment delivery, with a link to the pre-intervention survey. Of all staff emailed (sample size = 50), those who completed the pre-intervention survey within 4 weeks (*n* = 27) were recruited onto the study, resulting in a response rate of 54%. Due to four staff moving into non-clinical roles throughout the duration of the study, the final sample size was 23 at study completion. All participants had previously received pre-registration education on LEs.

### Data collection

Pre- and post-intervention online questionnaires were created using Survey Monkey^™^ software (San Mateo, CA).^21^ The questionnaires consisted of nine statements on pelvic radiotherapy LEs, each with 5-point Likert scale responses (Strongly Agree–Strongly Disagree). The statements were selected to determine participants’ perceptions on their current knowledge, training requirements, confidence and views on responsibility relating to pelvic LEs. Free-text comment boxes were provided to obtain a more detailed rationale for chosen responses. The questionnaires remained “open” for 4 weeks to account for staff absences.

10 participants were randomly selected to attend pre-intervention focus groups, split into two groups (*n* = 5&5). In order to reduce bias and potential reactivity due to the nature of the co-worker relationship between the principal researcher and the participants, all focus groups were conducted by a band 8 radiographer who was not involved in the study. Semi-structured interviews were conducted to ask pre-determined, open questions surrounding pelvic radiotherapy LEs knowledge, information provision and responsibility; this also provided the opportunity to address issues not originally considered and discuss topics raised by participants, thereby increasing research validity.^22^ With participants’ consent, all focus groups were recorded and moderated by the same band 8 radiographer to reduce bias.

The e-learning was accessible for 8 weeks. 4 weeks after the e-learning closed, post-intervention questionnaires were distributed and remained open for 4 weeks. Post-intervention focus groups were conducted with participants previously interviewed to gain longitudinal data. Participants were provided with an e-learning evaluation consisting of five open questions to appraise operability, asynchronous access, flexibility, effectiveness and preference on combined sessions. All responses were anonymised.

### Development of the e-learning intervention

The e-learning was developed using “Articulate” software (Articulate Global, New York)^23^ with support from the hospital’s Blended Learning Team and stored on the staff intranet. Content was derived from previously published data within the literature, including anecdotal evidence from patient interviews to demonstrate the impact of LEs on QoL. Staff responses to pre-intervention questionnaires highlighted a desire for further training on the incidence and management of pelvic LEs, therefore these topics were incorporated. Integration of NHS, NICE, SCoR and HCPC guidance documents highlighted the professional requirement to develop and utilise LEs knowledge to inform patients.

**Figure 1.**
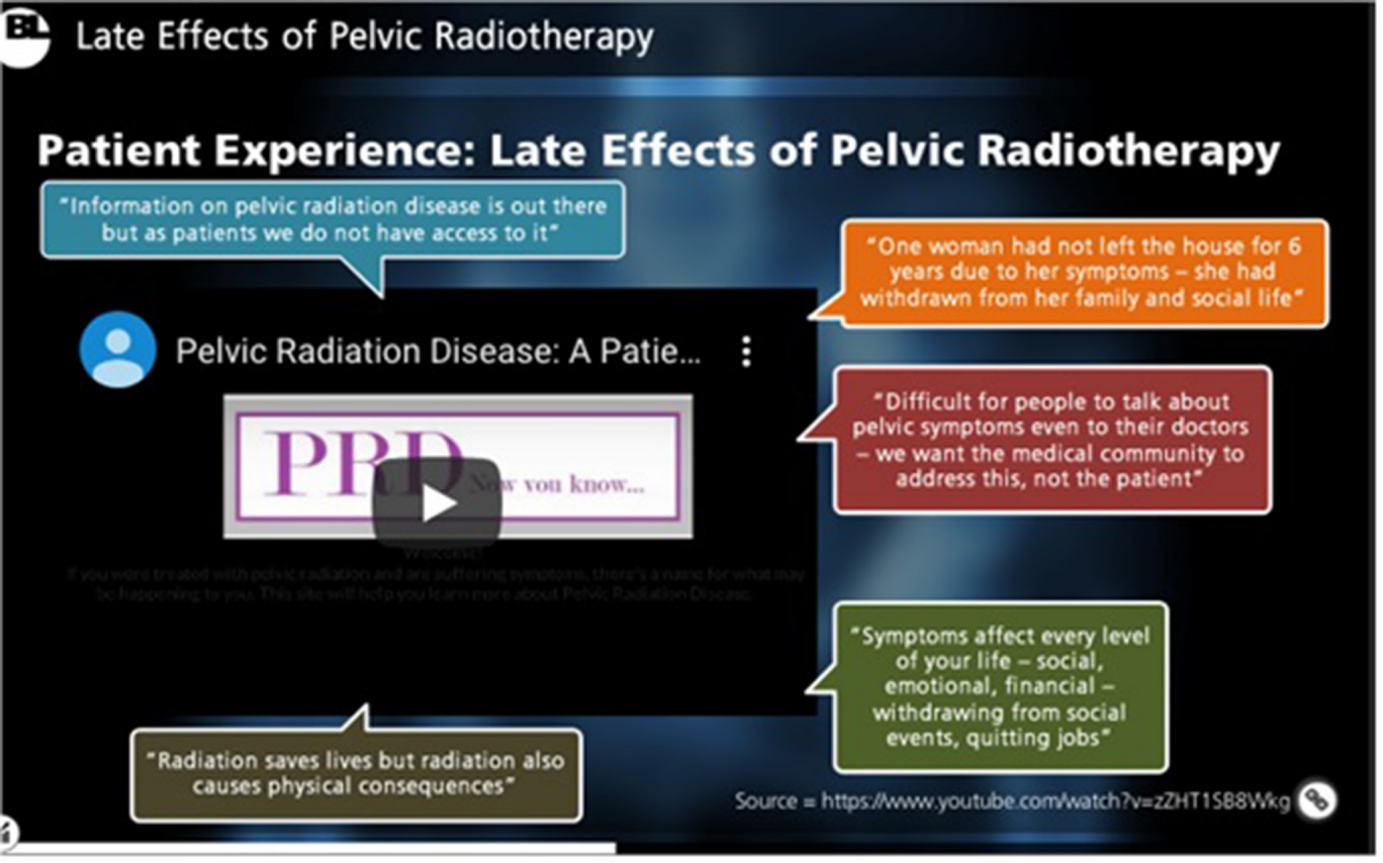
A sample of content included within the e-learning package: Patient Experience Video Clips. Patient experience videos were included with written quotes to emphasise the impact of LEs on patient QoL and provide an interactive format for enhanced learning. LE, late effect; QoL, quality of life.

**Figure 2.**
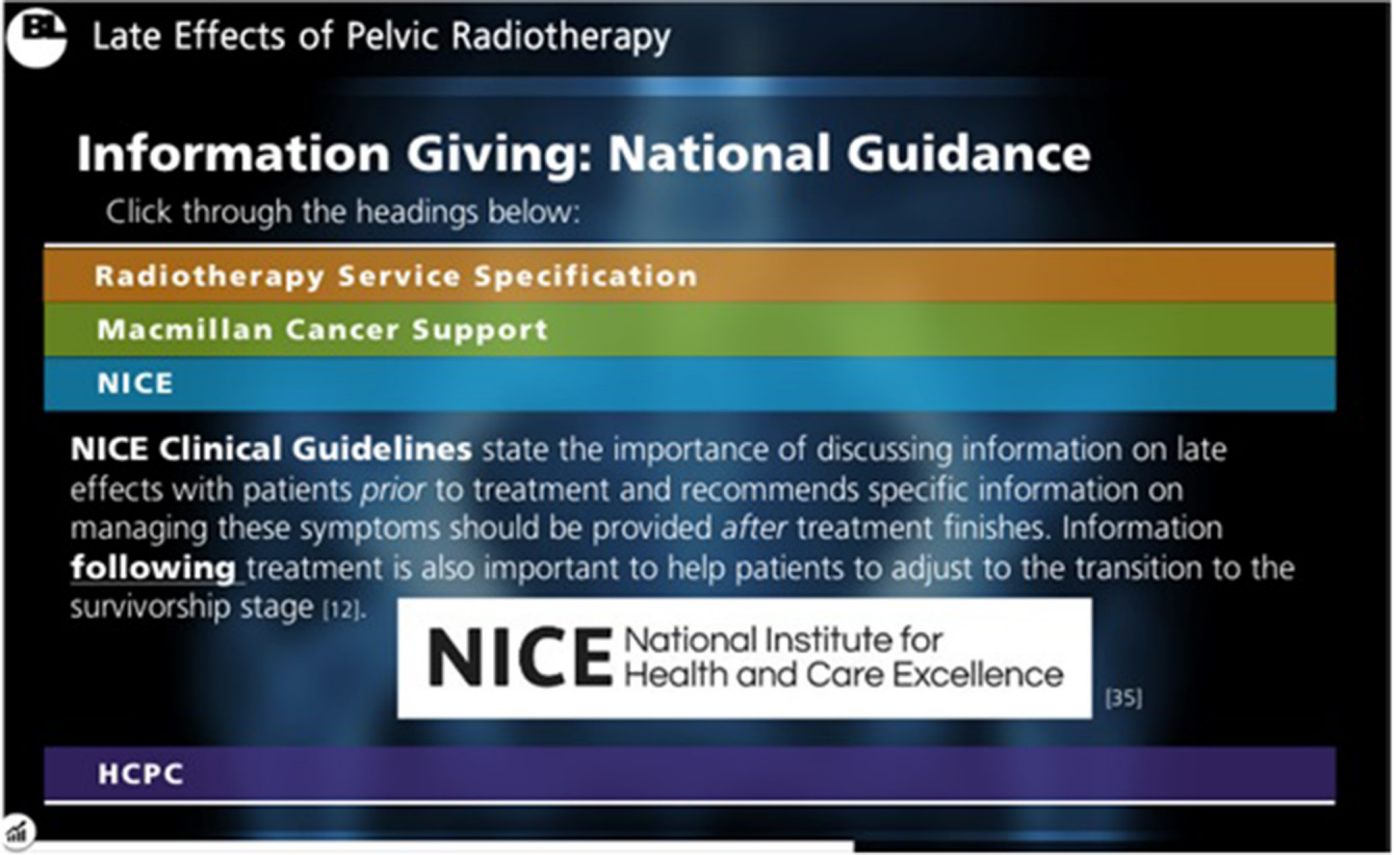
A sample of content included within the e-learning package: National Guidance on Information Giving. National guidance documents were included to highlight the professional requirement of providing LEs information to patients, provided in an interactive format to encourage information retention. LE, late effect.

**Figure 3.**
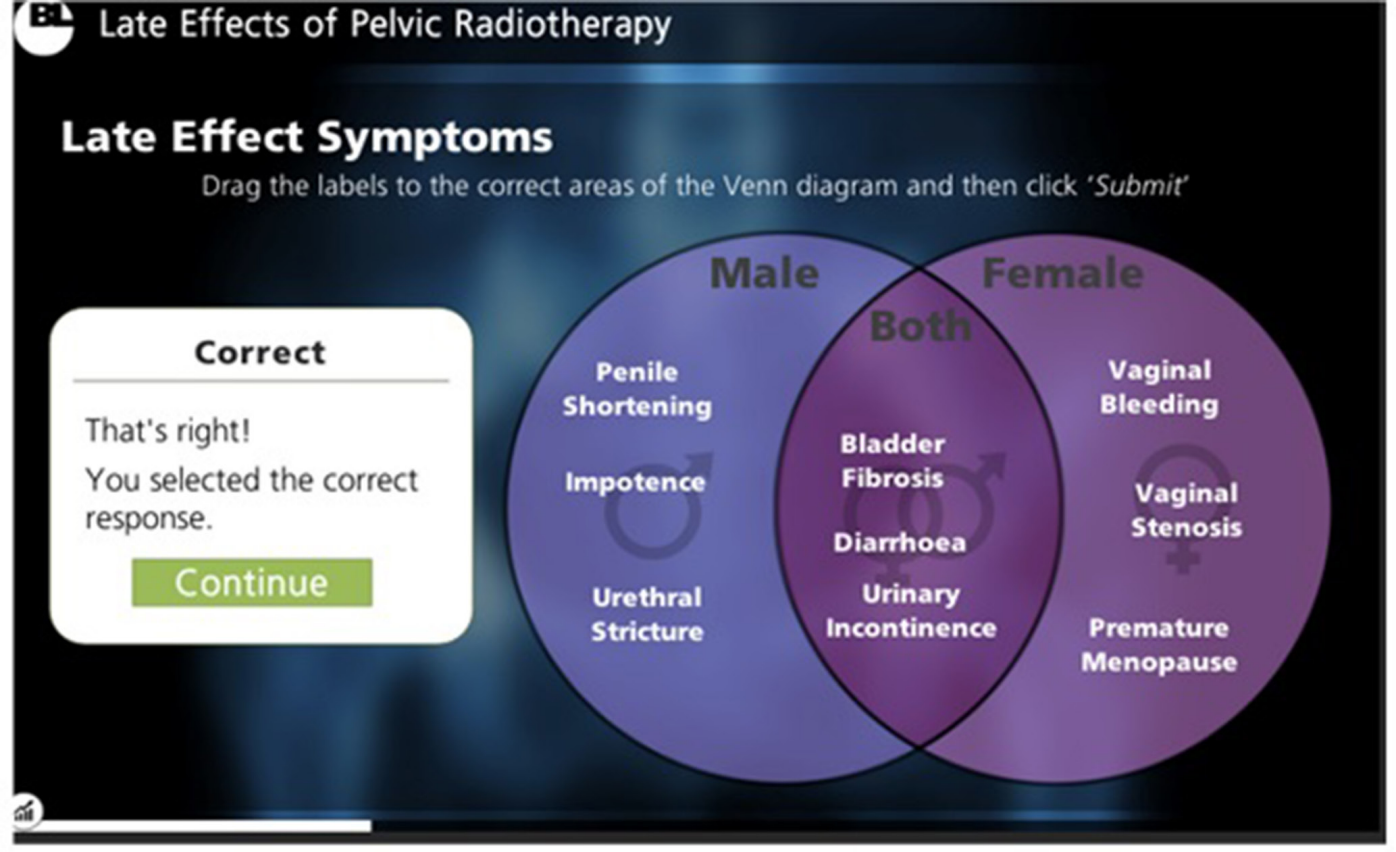
A sample of content included within the e-learning package: Interactive MCQs. MCQs were provided in a manner of different formats to stimulate learners and encourage information retention, *e.g.* using a Venn diagram with drag-and-drop answers. Learners were informed whether their answers were correct or incorrect, and provided with the solution for wrong answers. MCQ, multiple-choice question.

**Figure 4.**
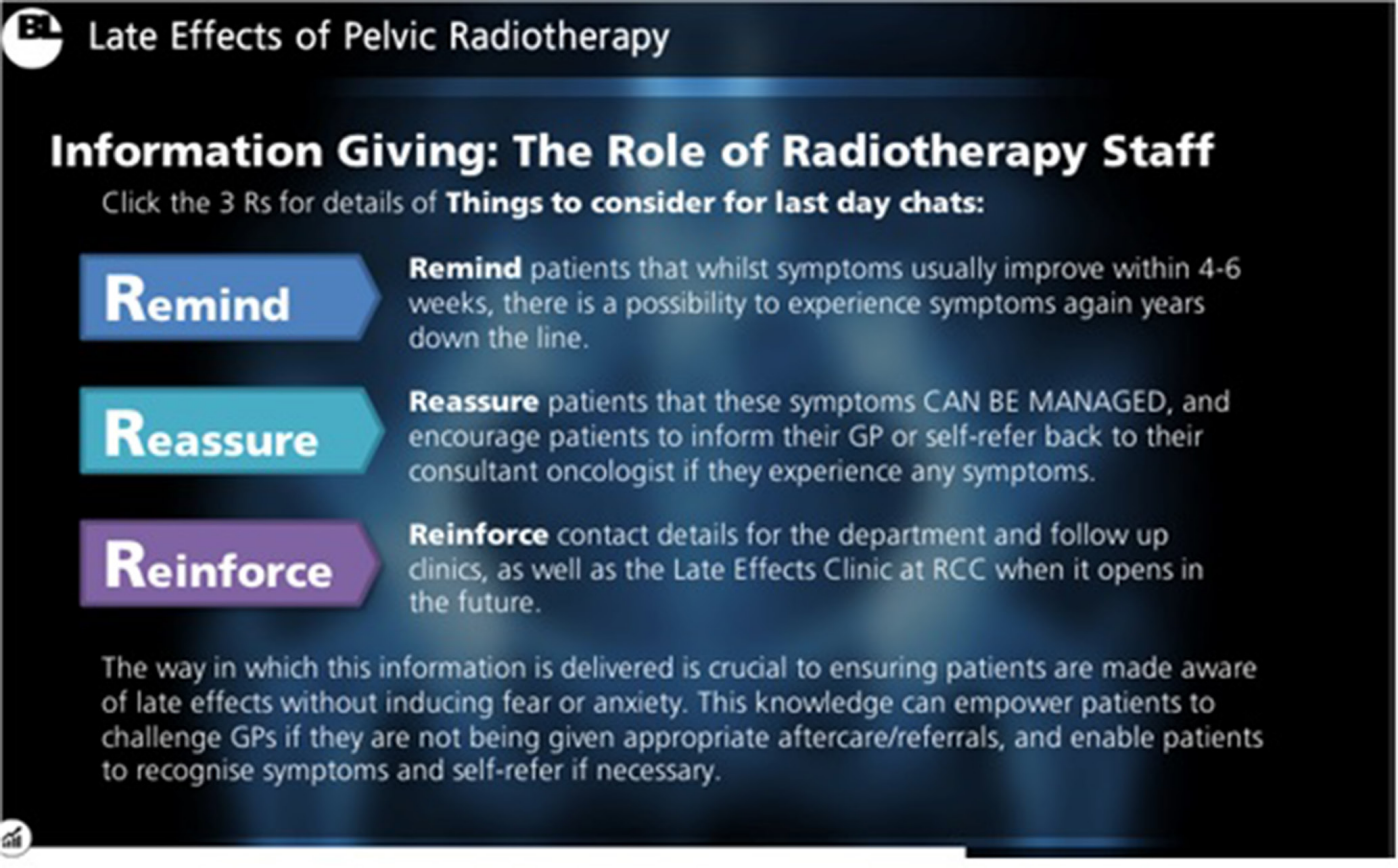
A sample of content included within the e-learning package: Things to consider for last day chats. Tips were provided for LEs information provision during conversations with patients on their final day of treatment. The “3 Rs” were created to encourage staff to remind patients of the possibility of LEs, reassure patients that these can be managed to prevent inducing worry or fear, and to reinforce contact information including details of the LEs clinic due to open to ensure patients receive optimal LEs management.

Multimodal material including sliding scales, “click-to-reveal” textboxes, videos and interactive MCQs ensured the package remained engaging (Figures 1–4). The asynchronous software allowed participants to pause and resume learning to account for time constraints within a busy clinical department.

Prior to dissemination, the e-learning was piloted by a band 8 radiographer and content discussed with a consultant radiographer to assess validity.

### Data analysis

Statistical analysis using a Wilcoxon signed-rank test was performed on paired pre- and post-intervention questionnaire responses using IBM SPSS Statistics v. 25 software (New York). A value of *p* < 0.05 was considered “significant”.

Thematic analysis of transcribed interview responses was conducted through “coding” of the data by two researchers. Analysis of large volumes of data is challenging, and requires an organised approach. First, data were organised into individual participant files; participants were assigned a non-identifiable ID number to ensure confidentiality and anonymity of responses. Transcripts were read in their entirety and memo notes made to highlight interesting, consistent and unique themes, words and descriptions.^24^ Initial noting ensured a growing familiarity with the transcripts, whilst descriptive commentary enabled participants’ words, phrases and explanations to be analysed, taking into consideration the language used. Conceptual comments were made, attempting to comprehend the participants’ meaning and described experiences. By evaluating connections and patterns from interview transcripts alongside free-text questionnaire comments, emergent themes were developed, reflecting matters of importance for participants. Given that this was a single-centre study with a sample size limited by the inclusion of “treatment delivery” radiographers only, fewer interviews were required to reach data saturation. The focus group sizes used here are consistent with that of similar qualitative studies; studies report the chance of identifying a new theme amongst six participants is >99% if that theme is shared across 55% of the wider study cohort.^25^ Based on the consistency of themes identified within the wider cohort’s questionnaire responses, the researchers were confident that the themes derived from the focus groups were representative of the larger study population’s views. The two researchers met throughout the analysis process to compare noting and commentary in order to establish agreement on themes described.

## Results

Four participants were excluded from the study following transition to non-clinical roles, resulting in a total sample size of 23 participants. The full qualitative results of the focus groups and questionnaires are presented as Supplementary Material 1.

### Thematic analysis

Five key themes were derived from transcribed focus group interviews:

1. Knowledge/Confidence.
2. Consent.
3. Professional responsibility.
4. Gaps within practice.
5. Time/Space.

#### Theme 1: knowledge/confidence

**Figure 5.**
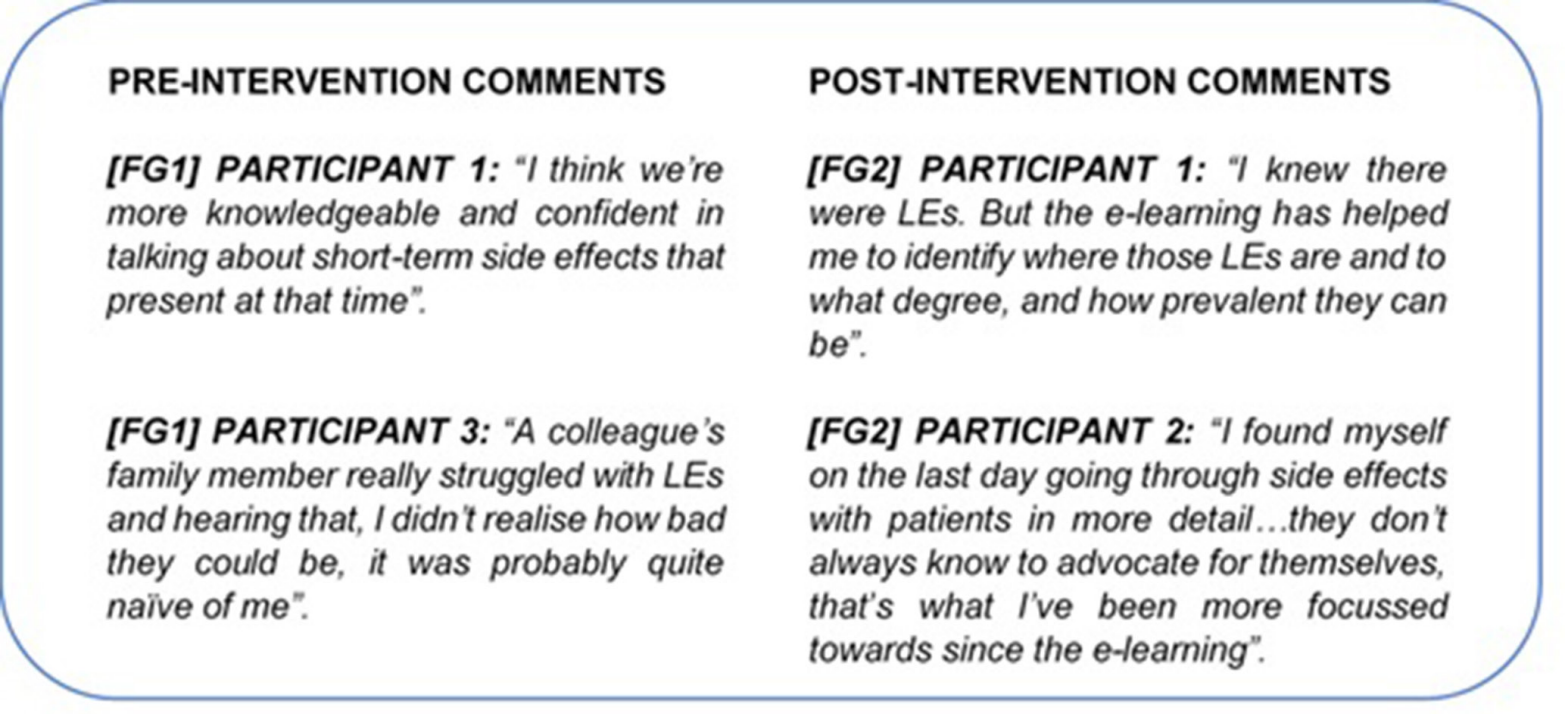
A sample of pre- and post-intervention comments from focus group interviews coded under the theme “Knowledge/Confidence” during thematic analysis. (Figure Suppl_1 in the Supplementary Material 1 for the full results).

The most significant difference noted between pre- and post-intervention focus group responses was participants’ perceptions of their knowledge and confidence surrounding pelvic radiotherapy LEs. Pre-intervention (*[FG1]*), several staff felt they lacked knowledge of LEs incidence, management and referral pathways, and admitted this led to them being less likely to discuss LEs with patients. All participants expressed a lack of confidence in discussing LEs with patients (Figure 5). Whilst several staff were aware of the physical LEs, it was clear that the realistic impact on patients’ QOL was not always apparent (Figure 5, FG1 participant 3).

Post-intervention (*[FG2]*), participants described an enhanced knowledge of pelvic LEs; several participants disclosed that this had already influenced their approach to information provision and benefitted patients (Figure 5, FG2 participants 1 & 2).

#### Theme 2: consent

When asked about responsibility in delivering LE information, participants discussed “informed consent” both pre- and post-intervention. Pre-intervention, participants expressed concerns that patients often present for radiotherapy with poor understanding of side-effects and discussed that LEs information must be given prior to treatment to obtain *informed* consent:

***[FG1] PARTICIPANT 2:*** *“…they should get to us day one and have had all the information”*.

Post-intervention, participants considered whether changes to the consent process were required to include more detailed information on LEs now survival rates have improved:

***[FG2] PARTICIPANT 5:*** *“…maybe that information needs to be adjusted to be in line with patients surviving longer”*.

Discussion of consent was much less extensive post-intervention, potentially due to shifts in staff perceptions regarding responsibility.

#### Theme 3: professional responsibility

Pre-intervention, a reluctance to accept professional responsibility for delivering LEs information was observed. Several staff associated this with the consultant during consent, however few participants did acknowledge their professional identity as HCPC registered radiographers:

***[FG1] PARTICIPANT 5:*** *“It’s the consultant’s [responsibility]…that should be part of a patient’s informed decision for consent”*.

***[FG1] PARTICIPANT 2:*** *“The consultant at consent…but then again us during radiotherapy because we’re actually beaming on…we need to make sure the patient’s got full knowledge of what’s going on”*.

However, following the e-learning there was a clear transition regarding the concept of responsibility, with participants more accepting of their role in delivering LEs information:

***[FG2] PARTICIPANT 2:*** *“I think it’s our responsibility as we’re the last point of contact before they go, to just refresh them”*.

**Figure 6.**
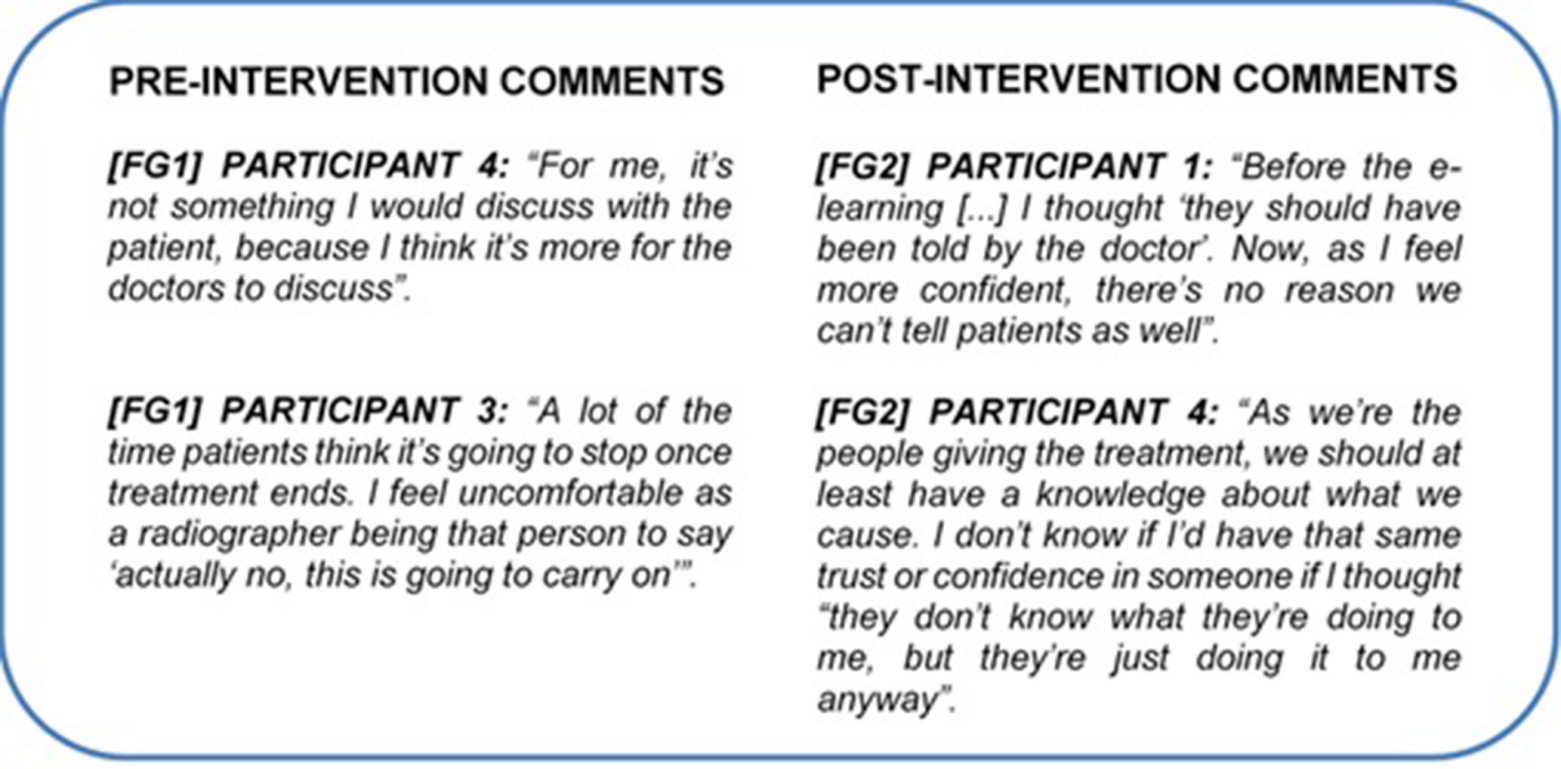
A sample of pre- and post-intervention comments from focus group interviews coded under the theme “Professional Responsibility” during thematic analysis. (Figure Suppl_3 in the Supplementary Material 1 for the full results).

Participants also linked this responsibility with LEs knowledge and confidence (Figure 6).

#### Theme 4: gaps within practice

Both pre- and post-intervention, participants acknowledged gaps within current practice. Several participants critiqued their own information provision and considered whether current information-giving practice instilled a sense of “false hope” in patients:

***[FG1] PARTICIPANT 3:*** *“We focus on short-term side-effects…no one ever talks about…‘actually these LEs are a risk down the line’”*.

**Figure 7.**
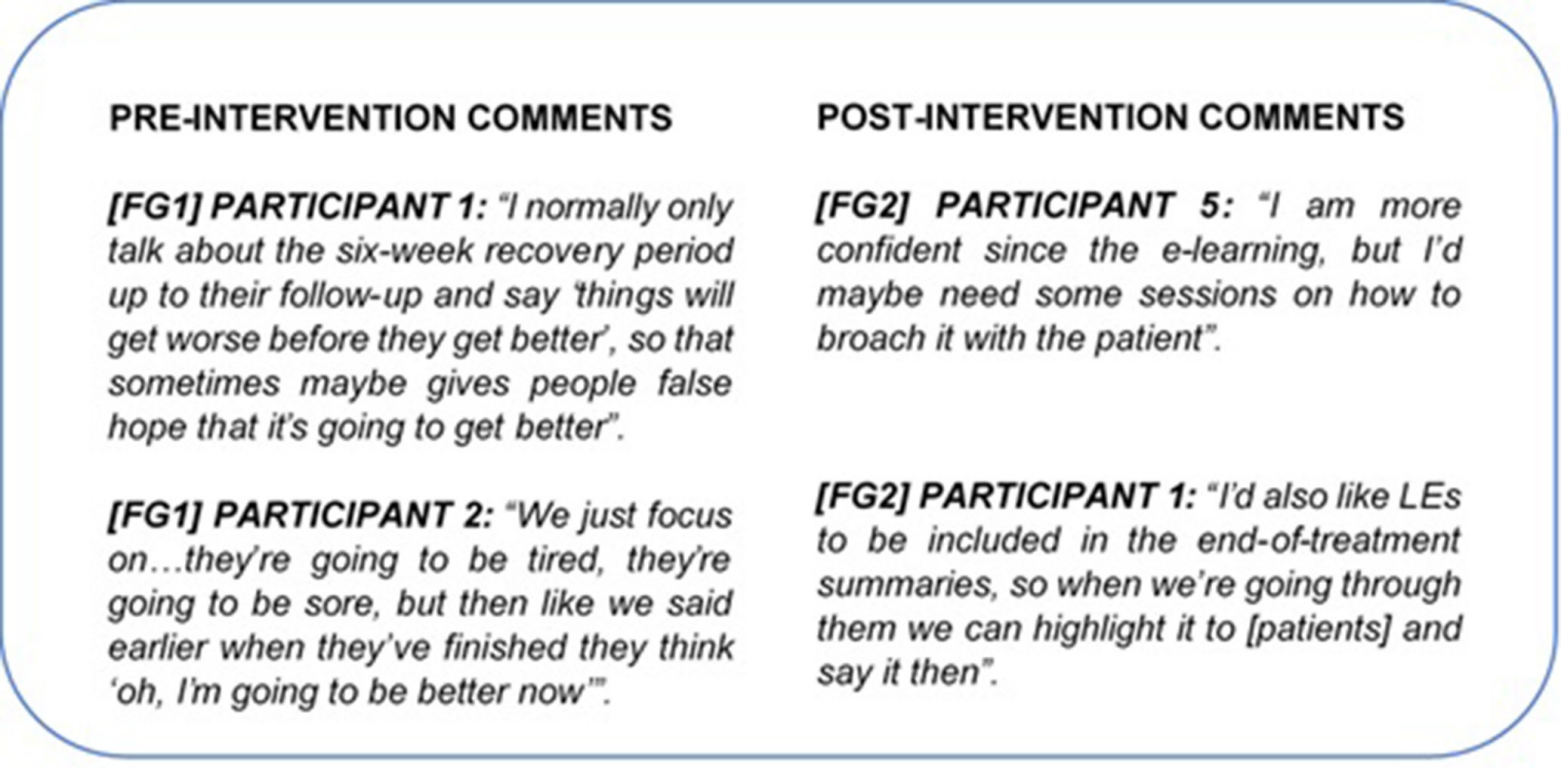
A sample of pre- and post-intervention comments from focus group interviews coded under the theme “Gaps within Practice” during thematic analysis. (Figure Suppl_4 in the Supplementary Material 1 for the full results).

Post-intervention, participants suggested improvements including additional training on discussing LEs with patients and amending “end-of-treatment summaries”, which were included in the e-learning to highlight the current lack of LEs information (Figure 7, FG2 participants 5 & 1).

#### Theme 5: time/space

Participants expressed that pressures within treatment delivery often leave insufficient time to provide a face-to-face chat to discuss side effects. This concept led to discussion of the feasibility of a “last day chat” at the end of treatment to provide such information in a private setting:

***[FG1] PARTICIPANT 3:*** *“…If you had an official sit-down chat like you did on the first day, you’d have time to go through LEs with the end-of-treatment summary”*.

### Questionnaire responses

Qualitative data from questionnaires revealed similar themes to those coded within interview responses. Pre-intervention comments (Table 1) revealed participants felt their knowledge and confidence in discussing LEs with patients could be improved, highlighting a requirement for post-registration training. Whilst several participants referred patients to their consultant to discuss LEs, others demonstrated a desire to provide this information themselves (Table 1).

**Table 1.** A sample of responses from pre-intervention questionnaire free-text comment boxes mapped against themes coded by thematic analysis (Table Suppl_1 in the Supplementary Material 1 for the full results)

*Theme*	Pre-intervention questionnaire free-text responses
*Knowledge/Confidence*	“Having been out of University for 4 years, I feel my knowledge could be refreshed” “Lack of training. Lack of time. Not really confident giving anything other than vague answers to patients” “I am confident to discuss some LEs that I have a greater understanding of, but unsure of others. I feel more training in this area would be very beneficial”
*Consent*	“Uncomfortable discussing as patients never really seem aware of them being long-term when they get to the radiotherapy stage” “Whenever I have divulged into LEs in first day chats, patients are sometimes unaware” “Patients must understand treatment side-effects in order to have informed consent”
*Professional responsibility*	“I often just offer advice on short-term side-effects and advise patients to speak to the doctor regarding longer term side effects” “I feel radiographers should be knowledgeable about all radiotherapy side-effects so they can prepare patients for the reality of life after treatment during last day chats” “I feel as a radiographer I should be able to discuss LEs in detail myself”
*Gaps within practice*	“I feel I very much concentrate on acute reactions rather than late effects unless these are listed on the consent form” “I definitely had training on late effects at University, but not in as much detail as the acute effects”
*Time/Space*	“I feel we generally have enough time to discuss these with patients during the normal working day”

LE, late effect.

Post-intervention free-text responses also aligned to participants’ interview comments; participants praised the e-learning for improving their awareness of pelvic radiotherapy LEs and demonstrated a willingness to take responsibility in delivering such information (Table 2).

**Table 2.** A sample of responses from post-intervention questionnaire free-text comment boxes mapped against themes coded by thematic analysis (Table Suppl_2 in the Supplementary Material 1 for the full results)

*Theme*	Post-intervention questionnaire free-text responses
*Knowledge/Confidence*	“I feel I am more aware of the symptoms of LEs of pelvic RT since completing the e-learning package” “I thought I had a good idea of these problems, after finishing the e-learning package I now realise the prevalence of these symptoms is much larger than I’d have guessed” “Although I feel more knowledgeable, I still don’t feel 100% confident in giving out specific statistics if asked by a patient, but I am more aware of mentioning to patients that there’s a chance of symptoms later on” “I am more confident/aware of the symptoms & happier to discuss the late effects with patients”
*Consent*	“A lot of information is discussed at the consent stage and patients do not always retain or understand this. I feel they could be informed more about this”
*Professional responsibility*	“I give out much more detailed and informed advice now because I am better informed on the subject. I also advise patients to advocate for themselves as the awareness of the problem is quite low in the community” “As an operator giving the radiation, I feel that the side-effects are my concern as well’ “As we deliver the treatment responsible for these LEs, we should be taking responsibility for them” “The treatment I deliver is directly responsible for late effects, therefore I am responsible for ensuring the patient has the information for future reference”
*Gaps within practice*	“Having done the e-learning, I don’t think patients receive the information they need for later down the line after finishing radiotherapy. With the introduction of last day chats and LEs information groups, I think it will be very beneficial”
*Time/Space*	“Sometimes time constraints don’t allow us to have detailed discussions with patients” “The e-learning has highlighted the importance of reiterating information regarding LEs at multiple time-points throughout the patient’s journey”

LE, late effect.

### e-Learning evaluation

Participants approved of the ease, flexibility and effectiveness of the e-learning in enhancing skills surrounding pelvic radiotherapy LEs (Table 3).

**Table 3.** A sample of participants’ open responses to the e-learning evaluation (Table Suppl_3 in the Supplementary Material 1 for the full results)

(1) How easy did you find the e-learning package to use? (*i.e.* the ease of use of the software itself, any technical issues experienced etc.)
The software was easy to use and follow. No technical issues. Fairly straightforward. Very easy, good instructions at the beginning and easy to follow prompts at each stage.
(2) Did you prefer being able to do the e-learning in your own time compared to scheduled face-to-face sessions, or would you have preferred scheduled sessions?
Yes, much easier being able to complete the training in my own time. Preferred doing it in my own time. I would personally like face-to-face sessions also, just because I take more information in this way. That being said, this package is really helpful for when we’re very busy and don’t have time to attend scheduled sessions.
(3) Did you find the e-learning package flexible? (*i.e.* being able to stop using the package and resume where you left off later on?)
Yes, although I did not need to do this, I liked that it was flexible and gave me the option to. Yes, this was helpful due to the limited time I have during the day to do training due to swapping of staff across shifts/lunch breaks etc. I did the package all in one go, but it is good to have that flexibility within our department.
(4) Did you find the e-learning effective, for example in improving your knowledge, skills or confidence surrounding the LEs of pelvic radiotherapy?
Yes, it was easy to understand and I feel I retained a lot of the information, which I can pass on to patients. Yes, I definitely feel I have gained knowledge and other skills from completing the e-learning. Yes, I feel more comfortable discussing late effects with patients since the e-learning. I think a yearly update (doing the package again) would help too.
(5) Do you think face-to-face sessions in addition to the e-learning would have improved your knowledge/skills/confidence in discussing LEs with patients?
Face to face sessions would help to consolidate learning, but with the time pressures we have it may be hard to achieve attending scheduled sessions. It would maybe be useful to further improve confidence though. I think it would be good to have a session to discuss what we have learned, and how to approach the topic with patients.

LE, late effect.

## Discussion

### Effectiveness of the e-learning intervention

We believe this study is the first of its kind, investigating the use of e-learning to improve pelvic radiotherapy LEs knowledge and awareness amongst therapeutic radiographers. Our novel e-learning proved successful in achieving these aims.

One of the most significant findings observed was participants’ perceived increase in their pelvic radiotherapy LEs knowledge. Thematic analysis revealed a recurring theme of “knowledge/confidence”, whereby a clear shift in participants’ views of their LEs knowledge was observed following the e-learning. Pre-intervention, several participants disclosed that they felt more knowledgeable about the short-term effects of treatment and often refrained from discussing LEs with patients. However, participants discussed how the e-learning resulted in changes to their information giving practice, benefitting patients:

***[FG2] PARTICIPANT 2:*** *“I found myself on the last day going through side-effects with [patients] in more detail [...] they don’t always know to advocate for themselves, and that’s what I’ve been more focussed towards since the e-learning”*.

Post-intervention questionnaire comments support this observation, with staff stating their knowledge had increased following the e-learning (Table 2). Similar findings are reported within the wider literature; statistically significant increases in staff knowledge (*p* < 0.001) have been observed following e-learning on patient safety, with knowledge levels remaining high for 12 months.^26^

The e-learning also increased participants’ awareness of the incidence and impact of LEs (Table 2), supporting recommendations made by UK charities. Jo’s Cervical Cancer Trust and the Pelvic Radiation Disease Association call for development of novel training to improve HCP knowledge, with recommendations to the SCoR to provide pedagogical opportunities on post-radiotherapy toxicities.^4^ Furthermore, Macmillan identified poor awareness of LEs as one of the “biggest barriers to improvement”, recommending solutions including e-learning.^27^

Following e-learning, staff confidence in discussing LEs with patients also increased; within post-intervention questionnaire comments, staff described feeling more confident and “*happier to discuss LEs with patients*” (Table 2). However, several participants appeared hesitant: *“although I feel more knowledgeable, I still don’t feel 100% confident”* (Table 2). Moreover, post-intervention thematic analysis indicated a requirement for additional training to further increase confidence:

***[FG2] PARTICIPANT 5:*** *“I am more confident[...]but I’d maybe need some sessions on how to broach it with the patient”*.

This concept is further supported by participants’ evaluation responses; several staff stated face-to-face training may be beneficial to improve confidence in discussing LEs with patients (Table 3). Research within the wider literature also advocates for additional sessions to support e-learning; studies comparing face-to-face teaching, e-learning and “blended” approaches combining the two methods for HCPs concluded the ‘blended’ approaches proved more effective than traditional or e-learning techniques alone (*p* < 0.001).^28,29^

Certainly, one of the most noteworthy findings of the study is the change in staff perceptions of responsibility. Pre-intervention, several participants expressed views (Table 1) that consultants are responsible for informing or reminding patients of pelvic radiotherapy LEs when obtaining informed consent:

***[FG1] PARTICIPANT 4:*** *“[…] it’s more for the doctors to discuss”*

***[FG1] PARTICIPANT 5:*** *“The consultant’s [responsibility]…that should be part of a patient’s informed decision for consent”*.

Reluctance of HCPs to accept responsibility for information provision due to the concept that it is “the consultant’s responsibility” or “should have been discussed during consent” is consistent with findings from similar qualitative studies in healthcare.^10,30^ This may suggest a reluctance to acknowledge that whilst radiotherapy aims to provide a beneficial outcome for patients, a degree of inevitable harm is caused that negatively impacts patients’ QOL.

However, following e-learning, thematic analysis and questionnaire comments (Table 2) demonstrated an increased acceptance of personal and professional responsibility:

***[FG2] PARTICIPANT 1:*** *“Before the e-learning [...] I thought ‘they should have been told by the doctor’. Now, as I feel more confident, there’s no reason we can’t tell patients as well”*.

Participants also reflected upon their professional responsibility as HCPC registrants; the HCPC Code of Conduct, Performance and Ethics states staff must provide patients with “*the information they want and need*”.^31^ Inclusion of such guidance documents within the e-learning aimed to highlight the professional requirement of radiographers to develop and utilise LEs knowledge when providing patient information (Figure 2).

Such changes in staff perceptions of the responsibility of radiographers was an impressive finding, and one of critical importance with the evolving roles of the radiotherapy profession.

### Wider impact of this research

Our novel e-learning package has now been disseminated to all NHS Hospitals within the Regional “Cancer Alliance”, a network aimed at improving cancer care locally.^32^ The e-learning has since been completed by surgeons, consultants and nurses amongst other professions treating pelvic radiotherapy patients. Through raising awareness across a variety of HCPs, there is potential for improved symptom recognition and diagnosis of LEs.

Furthermore, this e-learning was utilised to train nurses and dieticians at a novel “Radiotherapy LEs Clinic”. Therefore, not only has the e-learning demonstrated increased knowledge and awareness amongst therapeutic radiographers, but has since developed these areas amongst the wider oncology multidisciplinary team.

Despite participants all receiving pre-registration education at University, a significant lack of awareness of pelvic LEs was highlighted within this research. Recommendations from this study have led to the introduction of dedicated radiotherapy LEs modules on a novel MSc programme, providing post-registration education to further increase therapeutic radiographers’ knowledge.

### Clinical feasibility

The e-learning was appraised to determine its feasibility for clinical use. Participants approved of the e-learning to provide pelvic radiotherapy LEs training, describing the package as easy to use, flexible and effective in improving their skills (Table 3). Such findings are comparable to that reported by Shah & Stefaniak,^33^ whereby medical students described clinical e-learning as “easy, flexible, convenient and useful”.^33^

Such asynchronous training is crucial within fast-paced clinical departments, especially during the COVID-19 pandemic whereby remote access permits ongoing training for staff “shielding” at home.

Our study also demonstrates the potential to develop similar e-learning tools across other healthcare disciplines. For example, for radiotherapy professionals in departments looking to implement Magnetic Resonance (MR)-guided radiotherapy,^34^ or radiologists and diagnostic radiographers for training updates in dual energy CT imaging.^35^ For both, training can be asynchronous, overcoming the challenges of busy clinical workloads.

### Limitations

Participants evaluating their own knowledge produced subjective results and potential bias. “Reactivity” also posed a limitation, whereby participants may have behaved differently whilst being observed.^36^ Inclusion of staff of different years post-qualification meant those who have recently left education may have received different pre-registration LEs training to those who qualified several years prior. Furthermore, lack of a control group meant assumptions of cause–effect relationships may be less accurate. Finally, a small sample size was used, however due to the timeframe allocated for this MSc project a longer recruitment period was not feasible; a larger sample size in future audits of this e-learning will increase validity. The small sample size used here also limited the number of interviews required to reach data saturation. In future audits of the qualitative aspect of this study, data saturation will be considered prospectively and quantified prior to focus group interviews using the bootstrapping method, as described by Guest et al^25^.

## Conclusion

The novel, interactive e-learning package proved successful in achieving the study aims, demonstrating effectiveness in increasing knowledge and awareness amongst therapeutic radiographers within a single UK radiotherapy institution. Enhanced perceptions of professional responsibility in delivering patient information on pelvic radiotherapy LEs was also observed. Whilst the e-learning improved confidence amongst several participants, further training including “blended” pedagogical approaches may be required. Participants approved of the e-learning to provide LEs training in the clinical setting. In conclusion, the e-learning package developed here can be recommended due to its effectiveness and robust, asynchronous approach to HCP training.
